# 2431. Development and Implementation of a Risk Assessment Tool to Inform Quality Improvement in Infection Control among Vietnamese Hospitals

**DOI:** 10.1093/ofid/ofad500.2050

**Published:** 2023-11-27

**Authors:** Todd Pollack, Hoang Minh Nguyen, Thuy Thanh Pham, Tung Viet Cao, Ngai Kien Le, Van Anh Thi Dinh, Huong Van Tran, Ngoc Thi Bich Hoang, Ngoc Thi Bich Hoang, Thao Phuong Le, Preeti Mehrotra, Lisa A Cosimi, Dien Minh Tran

**Affiliations:** Beth Israel Deaconess Medical Center, Needham, Massachusetts; Beth Israel Deaconess Medical Center, Needham, Massachusetts; Beth Israel Deaconess Medical Center, Needham, Massachusetts; Vietnam National Children's Hospital, Hanoi, Ha Noi, Vietnam; Vietnam National Children's Hospital, Hanoi, Ha Noi, Vietnam; Vietnam National Children's Hospital, Hanoi, Ha Noi, Vietnam; Vietnam National Children's Hospital, Hanoi, Ha Noi, Vietnam; Vietnam National Children's Hospital, Hanoi, Ha Noi, Vietnam; Vietnam National Children's Hospital, Hanoi, Ha Noi, Vietnam; Beth Israel Deaconess Medical Center, Needham, Massachusetts; Beth Israel Deaconess Medical Center/Harvard Medical School, Boston, Massachusetts; Brigham & Women's Hospital, Boston, Massachusetts; Vietnam National Children's Hospital, Hanoi, Ha Noi, Vietnam

## Abstract

**Background:**

An estimated 70% of hospital-associated infections (HAIs) could be prevented if evidenced-based practices were consistently implemented. However, a recent study found that only 15% of health facilities globally met WHO infection prevention and control (IPC) minimum requirements. Strategies are needed to close this “Know-Do-Gap”, particularly in low-middle income countries. A community of practice (CoP) is a collaborative forum for sharing knowledge and approaches to improve care delivery. We established a CoP quality improvement initiative in Vietnam to spread IPC best practices in Vietnamese hospitals. We developed a risk assessment tool to assess current practices and capacity in the surveillance and prevention of HAIs and to inform planning for quality improvement efforts.

**Methods:**

A cross-sectional study was implemented in 4/2023 among 20 sub-national level hospitals in Vietnam prior to participation in the CoP. A facility assessment tool was developed with 3 domains: hospital characteristics, IPC capacity, and HAI prevention practices. Data was collected through self-reported questionnaires with data quality verification via online interviews with key staff from each facility. A scoring index was developed to stratify hospitals based on risk (Table 1 and 2), and a descriptive analysis was performed.

**Table 1:**
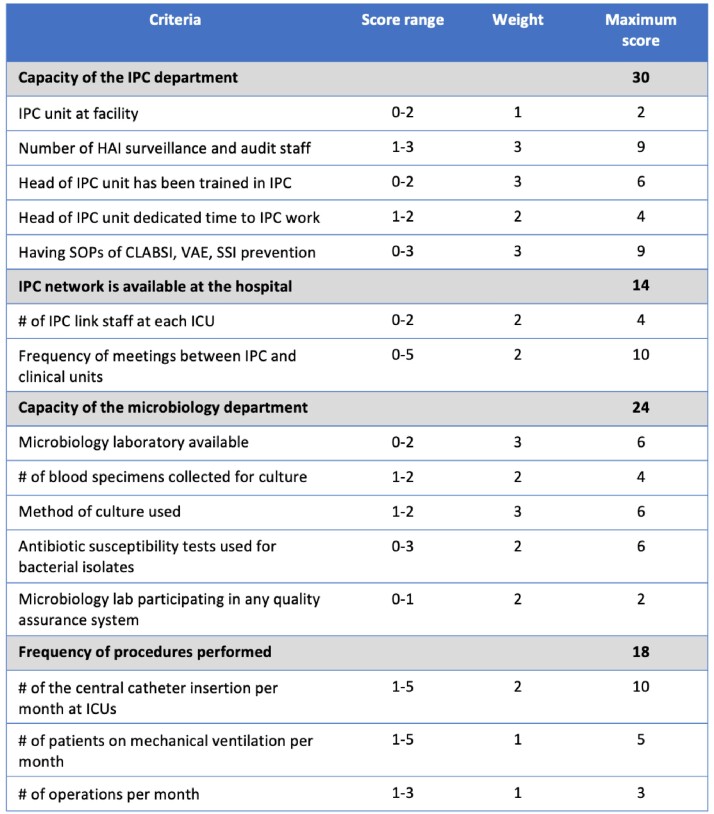
Hospital Infection Prevention and Control Capacity Scoring index. Abbreviations: IPC, infection prevention and control; HAI, hospital associated infections; SOP, standard operating procedures; CLABSI, central line associated blood stream infections; VAE, ventilator associated events; SSI, surgical site infections; ICU, intensive care unit. This table shows the weighed scoring index for assessing hospital infection control capacity. Each indicator was assigned a score from a range of options and a multiplier (1-3) was assigned based on the relative importance of the indicator.

**Table 2:**
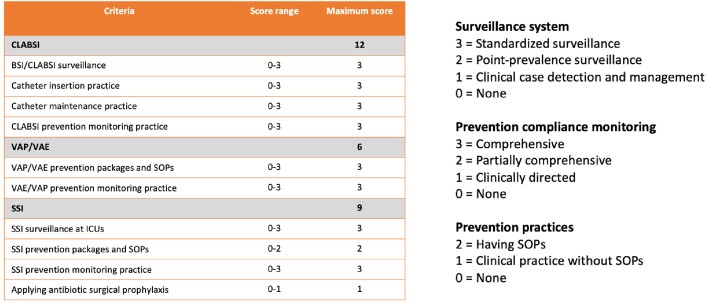
Hospital Infection Control Prevention Practices Scoring Index. Abbreviations: SOP, standard operating procedure; CLABSI, central line associated blood stream infections; BSI, bloodstream infections; VAE, ventilator associated events; VAP, ventilator associated pneumonia; SSI, surgical site infections; ICU, intensive care unit. This table shows the scoring index for assessing hospital associated infection prevention practices. Each indicator was assigned a score according to the associated legend.

**Results:**

Of the 20 hospitals, 12 reported at least 20 central line catheter insertions per month, 9 had at least 20 ventilated patients per month, and 19 performed more than 100 surgeries per month. Only 11 of 20 had any standardized operating procedures (SOPs) to guide prevention of HAIs and only 2 had SOPs for all 3 HAIs assessed. Less than half reported conducting regular meetings between IPC and clinical teams to improve IPC practices. Using the scoring index, differences in IPC practice and capacity were demonstrated among hospitals (Figure 1 and 2).

**Figure 1:**
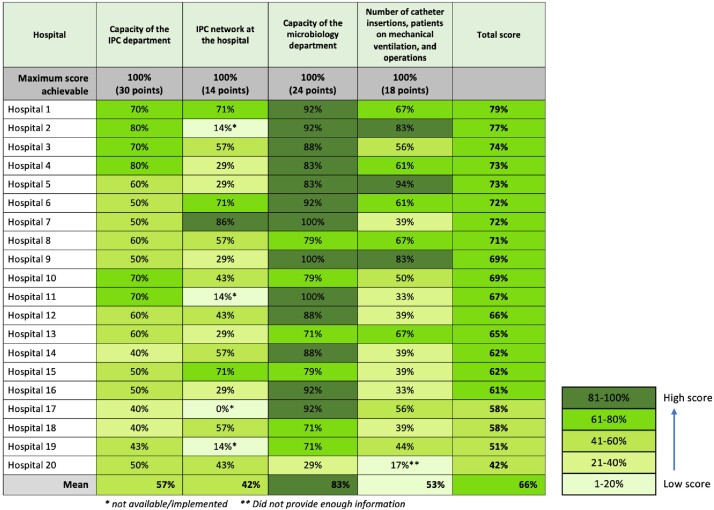
Infection Prevention and Control Capacity Scores, by Hospital

This figure is a heat map for visualizing the results of the assessment by domain and hospital. Scores for each domain were converted to percentages and assigned a color according to the legend.

**Figure 2:**
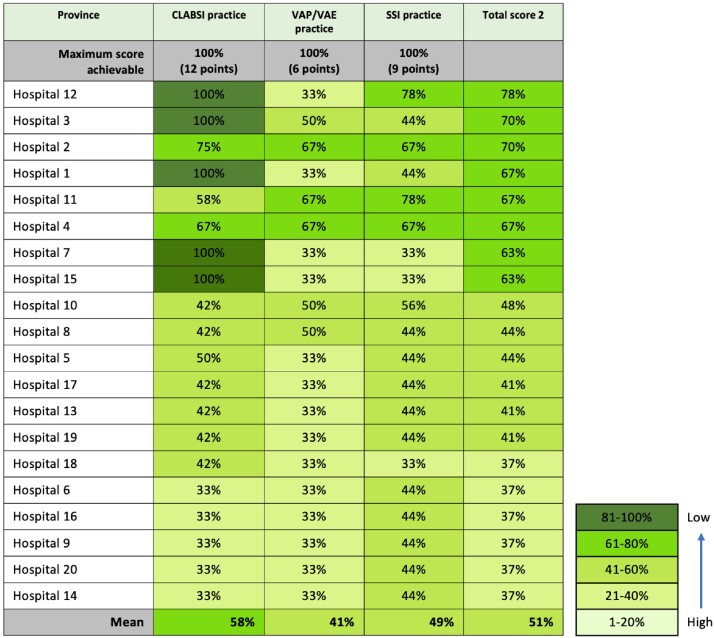
Infection Prevention Practices Scores, by Hospital

This figure is a heat map for visualizing the results of the assessment by domain and hospital. Scores for each domain were converted to percentages and assigned a color according to the legend.

**Conclusion:**

A risk assessment tool identified important gaps in IPC among participating hospitals in Vietnam, including the need for standardizing prevention practices and a lack of mechanisms for regular IPC-focused communication. Our results will guide allocation of resources for training and coaching within our CoP and will inform planning for interventions to spread evidenced-based practices within Vietnamese hospitals.

**Disclosures:**

**Todd Pollack, MD FIDSA**, AstraZeneca: Advisor for company hired by AZ|Gilead Sciences: Grant/Research Support

